# Comparative Genomic Analyses Provide Insight Into the Pathogenicity of *Metschnikowia bicuspidata* LNES0119

**DOI:** 10.3389/fmicb.2022.939141

**Published:** 2022-06-13

**Authors:** Hongbo Jiang, Jie Bao, Yuenan Xing, Xiaodong Li, Qijun Chen

**Affiliations:** Key Laboratory of Livestock Infectious Diseases in Northeast China, Ministry of Education, Shenyang Agricultural University, Shenyang, China

**Keywords:** *Metschnikowia bicuspidata*, genome annotation, comparative genomics, pathogenicity, *Eriocheir sinensis*

## Abstract

*Metschnikowia bicuspidata* is a globally distributed pathogenic yeast with a wide range of aquatic hosts. A new strain, *M. bicuspidata* LNES0119, isolated from the Chinese mitten crab *Eriocheir sinensis*, has caused a serious reduction in production and marked economic loss for the aquaculture industry in China. Therefore, the whole-genome sequence of *M. bicuspidata* LNES0119 was sequenced using Illumina and Oxford Nanopore technology; whole-genome annotation and comparative genomic analyses of this pathogen were performed as well. A high-quality genome of *M. bicuspidata* LNES0119 was 16.13 Mb in size, with six scaffolds and six contigs, and encoded 5,567 putative predicted genes. Of these, 1,467 genes shared substantial homology with genes in the pathogen–host interactions database. Comparative genomic analyses of three *M. bicuspidata* strains and one non-pathogenic yeast, *M.* aff. *pulcherrima*, showed 331 unique genes in *M. bicuspidata* LNES0119, 30 of which were putatively related to pathogenicity. Overall, we identified several meaningful characteristics related to pathogenicity and virulence that may play essential roles in the infection and pathogenicity of *M. bicuspidata* LNES0119. Our study will aid in identifying potential targets for further exploration of the molecular basis of the pathogenicity of *M. bicuspidata* as well as the therapeutic intervention of *M. bicuspidata* infection.

## Introduction

*Metschnikowia bicuspidata* (Metschnikoff) Kamienski (1899), which belongs to Fungi; Ascomycota; Saccharomycetales: Metschnikowiaceae: *Metschnikowia*, was first isolated from infected *Daphnia magna* by Metchnikoff (1884). Three strains are recognized according to their metabolic profile, biogeography, and habitat: *M. bicuspidata* var. *bicuspidata*, *M. bicuspidata* var. *californica*, and *M. bicuspidata var. chathamia* (Miller and Phaff, 1998).

*M. bicuspidata* is a globally distributed pathogenic fungus with a wide range of aquatic hosts. It exists in freshwater and marine environments worldwide, including in France, Romania, Russia, China, the United States, Canada, and even Antarctic waters (Bao et al., 2021). The hosts of *M. bicuspidata* include *Daphnia*, *Artemia*, snails, and the economically important freshwater prawn *Macrobrachium rosenbergii*, Chinese swimming crab *Portunus trituberculatus*, Chinese mitten crab *Eriocheir sinensis*, Chinese, grass shrimp (*Palaemonetes sinensis*) and chinook salmon (Moore and Strom, 2003; Wang et al., 2007; Bao et al., 2021; Cao et al., 2022). Among these hosts, the *Daphnia*-*M. bicuspidata* system has long been considered a model system in ecology and evolutionary biology and has been used as a model organism in the exploration of host-parasite theory. For example, *M. bicuspidata* is considered to be highly virulent and has been suggested to have affected the evolution of *D. dentifera* populations in Bristol Lake, United States (Duffy et al., 2008). In aquaculture, many economically important animals have been infected by *M. bicuspidata*, resulting in a decline in production and marked economic loss. For example, an outbreak of *M. bicuspidata* in Taiwan from May 2001 to December 2003 resulted in cumulative mortality rates of 20–95% in *M. rosenbergii* (see Chen et al., 2003, 2007), 40–60% in *P. trituberculatus* in Zhoushan, Zhejiang Province, China, from 2002 to 2006 (Shi et al., 2008), over 20% in *E. sinensis* in Panjin, Liaoning Province, China, from 2018 to 2019 (Bao et al., 2021), and 34.5% in larval chinook salmon fed on infected *Artemia* in California, United States (Moore and Strom, 2003). The aquatic hosts were infected directly by ingesting *M. bicuspidata* spores or indirectly by consuming diseased individuals, after which the spores in the body cavity began producing hyphae and adhered to nearby surfaces, producing conidia that rapidly increased in abundance within the host (Merrill and Cáceres, 2018; Jiang et al., 2022; Sun et al., 2022).

In 2019, we isolated the *M. bicuspidata* LNES0119 strain from infected *E. sinensis* from ponds in Panjin city, Liaoning Province, and using the D1/D2 domain of the 26S rDNA, we identified it as the pathogen causing milky disease in Chinese mitten crab (Bao et al., 2021), which is an economically important freshwater crustacean in China. This epidemic showed the characteristic symptoms of milky hemolymph and death due to organ failure. A milky disease epidemic was also detected in Xinjiang, Heilongjiang, and Jilin provinces, and the mortality rates in 2021 were even higher than that in 2020, which had seriously affected the Chinese mitten crab industry (Sun et al., 2022). Therefore, in this study, the whole-genome sequence of the strain *M. bicuspidata* LNES0119 was sequenced using Illumina and Oxford Nanopore technology. The whole-genome annotation and comparative genomic analyses with two *M. bicuspidata* stains and one non-pathogenic *M.* aff. *pulcherrima* stain were carried out to explore the genes or gene families associated with pathogenicity, our analyses will provide genomic resources for future exploration of the pathogenesis and molecular basis of the pathogenicity of *M. bicuspidata*.

## Materials and Methods

### Yeast Strain and DNA Isolation

The *M. bicuspidata* strain LNES0119 was isolated from a diseased Chinese mitten crab in Panjin city. The strain was stored at −80°C in 20% glycerol (v/v), incubated on Rose Bengal agar medium, cultured for 48 h at 28°C, and transferred onto a new medium, followed by further culturing for 48 h before collecting the yeast. Genomic DNA was extracted using the Qiagen Genomic-Tip 100/G Kit (Qiagen, Hilden, Germany) according to the manufacturer’s instructions. DNA quality was assessed via 0.35% agarose gel electrophoresis and quantified using NanoDrop and Qubit Fluorometer 3.0 (Thermo Fisher Scientific, Waltham, MA, United States).

### Library Construction, Genome Sequencing, and Assembly

The *M. bicuspidata* strain LNES0119 was first surveyed using Illumina NovaSeq 6000 platform TruSeq libraries (150 bp paired end reads, insert size of 350 bp) and then sequenced using the long reads Oxford Nanopore sequencing platforms at Beijing Biomarker Technologies (Beijing, China). Large fragments of DNA (DNA fragments > 20 kb) were first collected using the BluePippin Size-Selection system (Sage Science, Beverly, MA, United States). Next, the DNA sequencing library was constructed, including DNA repair, end-prep, and adapter ligation, and clean-up steps were performed with the ligation kit SQK-LSK109 (Oxford Nanopore Technologies, Oxford, United Kingdom) according to the manufacturer’s instructions. The final product was quantified using a Qubit fluorometer (Thermo Fisher Scientific, Waltham, United States) and loaded into the PromethION flow cell, and real-time single-molecule sequencing was performed according to the manufacturer’s instructions (Oxford Nanopore Technologies, Oxford, United Kingdom). The Nanopore reads were base-called from the raw Fast5 files using Albacore (Oxford Nanopore Technologies). Fastq reads were filtered with a quality value of Q > 7. The filtered subreads were first corrected and assembled using Canu version 1.5 (Koren et al., 2017) and Wtdbg version 2.2 (Ruan and Li, 2020). Pilon version 1.22 (Walker et al., 2014) was further applied to correct any sequencing errors caused by using second-generation results, and a genome sequence with a higher accuracy was obtained. BUSCO v.2.0, Simão et al. (2015) was used to assess the completeness of the assembled genome based on the Benchmarking Universal Single-Copy Orthologs (BUSCOs) for Fungi (fungi_odb9) dataset.

### Genomic Prediction and Genome Annotation

For prediction of repeat sequences, LTR_FINDER v1.05 (Xu and Wang, 2007), MITE-Hunter (Han and Wessler, 2010), RepeatScout v1.0.5 (Price et al., 2005) and PILER-DF v2.4 (Edgar and Myers, 2005) software were used to construct a *de novo* repeats library of the *M. bicuspidata* LNES0119 genome using a combination of homology-based and *de novo* approaches. PASTEClassifier (Wicker et al., 2007) was used to classify the database, and then combined with the Repbase database (Jurka et al., 2005) to obtain the final repeat library, which was annotated using RepeatMasker V4.0.6 software (Chen, 2004). Transfer RNAs (tRNAs) were predicted by tRNAscan-SE 2.0 (Lowe and Eddy, 1997), whereas ribosomal RNAs (rRNAs) and other non-coding RNAs (ncRNAs) were predicted using Infernal 1.1 (Nawrocki and Eddy, 2013) based on Rfam database (Nawrocki et al., 2015).

Protein-coding genes in the *M. bicuspidata* LNES0119 genome were predicted using a combination of *ab initio* prediction, homology-based protein prediction, and transcriptome-based prediction. For *ab initio* prediction, Genscan (Burge and Karlin, 1997), Augustus V2.4 (Stanke and Waack, 2003), GlimmerHMM V3.0.4 (Majoros et al., 2004), GeneID V1.4 (Blanco et al., 2007), and SNAP (version 2006-07-28) (Korf, 2004) were used. For homologous protein-based prediction, protein sequences from *M. bicuspidata* were downloaded from the NCBI database and then used for gene annotation by aligning against the *M. bicuspidata* LNES0119 genome using GeMoMa V1.3.1 (Keilwagen et al., 2016). For transcriptome-based prediction, HISAT v2.0.4 and StringTie v1.2.3 (Pertea et al., 2016) were used to assemble and map our referenced RNA-seq data. TransDecoder v2.0,^1^ GeneMarkS-T v5.1 (Tang et al., 2015) and PASA v2.0.2 (Campbell et al., 2006) were used to predict assembled UniGene sequences. Finally, EVM V1.1.1 (Campbell et al., 2006) was used to integrate the above three prediction results, and PASA v2.0.2 was employed for modification.

Functional annotations of all predicted gene models were compared with the following databases: Eukaryotic Orthologous Groups (KOG), Kyoto Encyclopedia of Genes and Genomes (KEGG), NCBI non-redundant protein sequences (Nr), Swiss-Prot, and TrEMBL using BLASTP with an e-value cutoff of 1e-5. Blast 2GO (Conesa et al., 2005) was used for gene annotation based on gene ontology (GO). Potential pathogenicity-related proteins were identified using the pathogen–host interactions (PHI) database (Winnenburg et al., 2006). HMMER (Eddy, 1998) was used to detect transporters based on the Transporter Classification Database (TCDB) (Saier et al., 2006) and to annotate the predicted protein sequences using the Pfam database (Finn et al., 2016). Carbohydrate-active enzymes (CAZymes) were actualized using the CAZy database as well as the dbCAN CAZyme HMM database (Yin et al., 2012). Secreted proteins were predicted by Signal P 4.0 (Petersen et al., 2011) and TMHMM (Krogh et al., 2001), and the proteins containing signal peptides and without a putative transmembrane structure were regarded as secreted proteins. Potential effector proteins were predicted using EffectorP (Sperschneider et al., 2016) with a probability threshold of 0.5.

### Comparative Genomics Analysis

Two pathogenic strains of *M. bicuspidata* Baker 2002 (PRJNA421585) and *M. bicuspidata* NRRL YB-4993 (PRJNA207846), and one non-pathogenic *M.* aff. *pulcherrima* APC 1.2 (PRJNA508581) species were selected for comparison of their genomic data with the genomic data of *M. bicuspidata* LNES0119 (Table 1). Collinearity analysis was performed using the MCScanX software (Wang et al., 2012). Gene orthology analysis was conducted using OrthoMCL (Li et al., 2003), and the gene families were analyzed, including the gene families specific to the strains, the gene families common to all species, and the gene families with a single copy of each strain. Functional annotation of gene families was performed using the Pfam database (Finn et al., 2016). For comparative analysis of the CAZymes, data from *M. bicuspidata* LNES0119 and 12 other yeast strains were processed using the dbCAN CAZyme HMMs database.

**TABLE 1:** Genome characteristics of four *Metschnikowia* species strains.

	*M. bicuspidata* LNES0119	*M. bicuspidata* Baker 2002	*M. bicuspidata* NRRL YB-4993	*M.* aff. *pulcherrima* APC 1.2
Genome size (Mb)	16.13	10.95	16.06	15.80
Scaffolds	6	478	48	7
Contigs	6	488	582	7
N_50_	3,357,032	132,937	62,344	2,688,662
GC (%)	47.65	51.1	47.9	45.89
Coverage	175.82 X	100 X	16 X	254.0 X
Number of genes	5,567	4,890	6,090	6,018
Number of predicted proteins	6,478	4,777	5,838	5,800
Sequencing technology	Nanopore	Illumina	454, Illumina	PacBio
Location	China	Michigan, United States	Peoria, IL United States	Switzerland
Lifestyle/host	Single-cell/crabs	Non-free-living/daphnia	Single-cell/brine shrimp	Single-cell/flowers

*M. bicuspidata Baker 2002 was isolated from an infected population of the water flea Daphnia dentifera. M. bicuspidata NRRL YB-4993 is an aquatic yeast that has been reported to infect freshwater prawns and brine shrimps and to cause mortality when infected shrimps are fed to salmon. M. aff. pulcherrima APC 1.2 was isolated from apple flowers as antagonist.*

## Results

### Genome Sequencing, Assembly, and Assessment

A total of 16.13 Mb of sequence data (175.82 X coverage), with six scaffolds and six contigs, were obtained from the whole genome of the *M. bicuspidata* strain LNES0119 (Table 2). Analysis of the length distribution of long and high-quality reads yielded a high-quality library (Supplementary Figure 1). The scaffold lengths of N50 and N90 were 3,357,032 and 2,548,385 bp, respectively, and the maximum length was 3,791,280 bp (Table 2). The average GC content was 47.65%. The number and average length of the predicted coding genes were 5,567 and 9,595,085 bp, respectively. The total length of the repeat sequences was 475,761 bp, accounting for 2.95% of the genomic length. These contained Class II transposable elements, including four Helitrons and 26 terminal inverted repeats, Class I or the retroelements including 359 LINE, 60 LTR/Copia, 153 LTR/Gypsy, and 426 non-LTR/LINE (Supplementary Table 1). With respect to RNAs, 88 rRNAs, 306 tRNAs, and 89 other ncRNAs were predicted (Supplementary Table 2). The genome was estimated to be 99.3% complete with 288 complete BUSCOs, one fragmented BUSCO, and one missing BUSCO from a total of 290 BUSCO groups. Overall, these results indicate that the genome of our *M. bicuspidata* strain was characterized as high-quality, complete, and accurate.

**TABLE 2:** Genome characteristics of the *Metschnikowia bicuspidata* strain LNES0119.

Assembly and characteristics	
Genome size (Mb)	16.13
N50 Length (bp)	3,357,032
N90 Length (bp)	2,548,385
Max length (bp)	3,791,280
GC (%)	47.65
Coverage	175.28X
Scaffolds number	6
Gap number	0
BUSCO (% complete)	99.3%
Number of genes	5,567
Mean number of exons	1.17
Mean number of introns	1.16
Average length of exons (bp)	1,434.08
Average length of introns (bp)	264.11
Number of predicted proteins	6,478

### Genome Annotation

**FIGURE 1:**
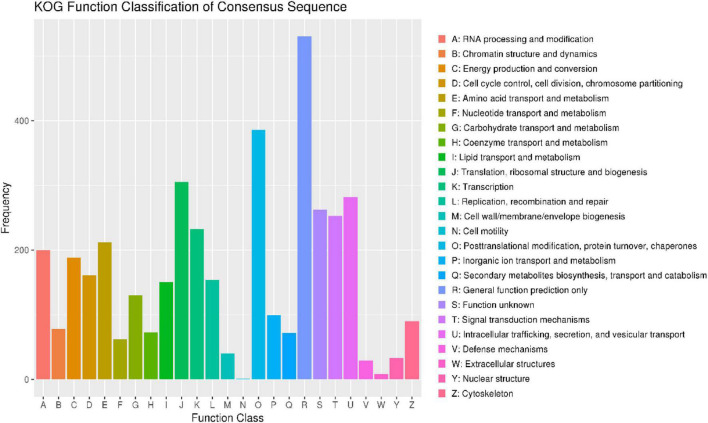
Clusters of orthologous groups of proteins (KOG) function classification of proteins in *Metschnikowia bicuspidata* LNES0119.

To annotate the function of the predicted genes in the *M. bicuspidata* LNES0119 genome build, 5,567 predicted genes were annotated using multiple public databases: Nr, GO, KEGG, KOG, TCDB, Pfam, TrEMBL, and Swiss-Prot databases are shown in Supplementary Table 3. According to the GO database, 3,175 predicted proteins that accounted for 57.03% of the entire genome were primarily distributed in three categories: cellular components, molecular function, and biological process (Supplementary Figure 2 and Supplementary Table 3). NCBI KOG mapping predicted that 3,590 genes (64.49%) were assigned to KOG categories (Figure 1). The most enriched KOG category is “General functional prediction only” (530), followed by “Posttranslational modification, protein turnover, chaperones” (386), “Translation, ribosomal structure and biogenesis” (305), “Intracellular trafficking, secretion, and vesicular transport” (282), “Function unknown” (262), and “Signal transduction mechanisms” (253). A total of 2,869 genes were annotated in the KEGG database and were separated into three specific categories: genetic information processing, environmental information processing, metabolism, and cellular processes (Supplementary Figure 3 and Supplementary Table 3). Among them, “Ribosome” (113) and “Biosynthesis of amino acids” (104) contained the largest number of genes.

**FIGURE 2:**
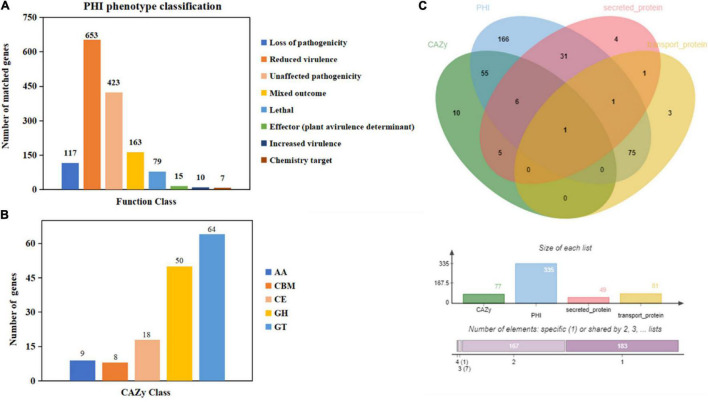
Gene annotation and gene prediction of *M. bicuspidata* LNES0119. **(A)** Genes were annotated and classified in the PHI database. Bars in different colors represent different PHI function classes, and lengths represent the number of genes. **(B)** Genes were annotated and classified in the CAZy database. Bars in different colors represent different CAZy categories, and lengths represent the number of genes. GH, glycoside hydrolase; GT, glycosyltransferase; CE, carbohydrate esterase; AA, auxiliary activity; CBM, carbohydrate-binding module. **(C)** Venn diagram showing the overlap of PHI-homologs and secretory proteins with transport protein and CAZymes.

Moreover, 1,467 genes were annotated in the PHI database into different categories, which predicted protein function during host infection (Figure 2A and Supplementary Table 4). Of these genes, 653 genes were annotated to reduced virulence, 432 genes were annotated as unaffected pathogenicity, and there were 163 genes annotated as mixed outcomes. A total of 117 and 79 genes were annotated as pathogenic loss and lethal factors, respectively, whereas seven and 15 genes were annotated as chemistry targets and effectors (plant avirulence determinant), respectively. Only 10 genes were annotated as having enhanced virulence.

There were 149 genes annotated in the CAZy database, which were divided into five categories, of which 64 genes (42.95%) annotated as glycosyl transferases (GTs), 50 genes (33.55%) annotated as glycoside hydrolases (GHs), 18 genes (12.08%) annotated as carbohydrate esterases (CEs), nine genes (6.04%) annotated as auxiliary activities enzymes (AAs), and eight genes (5.36%) annotated as carbohydrate-binding modules (CBMs) (Figure 2B).

The Venn map of *M. bicuspidata* LNES0119 was obtained according to the annotation results of CAZy, PHI, transported protein, and secreted protein (Figure 2C). A total of 291 proteins with signal peptides and 1,066 with transmembrane structures were detected in the genome of *M. bicuspidata* LNES0119. Of these, 158 were identified as potential secreted proteins (including 18 with putative effectors), of which 41 were annotated using the PHI database, and these secreted proteins were primarily cell wall proteins and hydrolytic proteins (Table 3 and Supplementary Table 5). In addition, 62 CAZymes protein clusters (including 64 genes) were annotated to PHI (Figure 2C), which were enriched in protein O-linked glycosylation (GO:0006493), glucan catabolic process (GO:0009251), and protein mannosylation (GO:0035268). The Venn diagram showed that there was only one protein cluster (multicopper oxidases) that was annotated to CAZymes, PHI, transported protein, and secreted protein, which belonged to iron ion homeostasis (GO:0055072).

**TABLE 3:** Secretory proteins associated with pathogenicity.

Secreted_protein ID	Pfam annotation	PHI-base entry	Phenotype of mutant
EVM0001456.1	Cellulase (GH5)	PHI:323	Reduced virulence
EVM0005284.1	Homoserine dehydrogenase	PHI:323	Reduced virulence
EVM0001841.1	GH65	PHI:3076	Reduced virulence
EVM0005201.1	Glucanosyl transferase	PHI:33	Reduced virulence
EVM0001397.1	Thioredoxin	PHI:2644	Reduced virulence
EVM0001511.1	Thioredoxin	PHI:2644	Reduced virulence
EVM0005208.1	Thioredoxin	PHI:2644	Reduced virulence
EVM0000597.1	Subtilase family	PHI:2117	Reduced virulence
EVM0002445.1	Subtilase family	PHI:2117	Reduced virulence
EVM0004644.1	Ribonuclease T2 family	PHI:811	Reduced virulence
EVM0001314.1	Multicopper oxidase	PHI:2700	Reduced virulence
EVM0005103.1	Lysophospholipase catalytic domain	PHI:105	Reduced virulence
EVM0000180.1	Lipase (class 3)	PHI:432	Reduced virulence
EVM0005523.1	Hsp70 protein	PHI:2058	Reduced virulence
EVM0002216.1	Eukaryotic aspartyl protease	PHI:17	Reduced virulence
EVM0002655.1	Eukaryotic aspartyl protease	PHI:17	Reduced virulence
EVM0004851.1	Eukaryotic aspartyl protease	PHI:17	Reduced virulence
EVM0003594.1	Eukaryotic aspartyl protease	PHI:17	Reduced virulence
EVM0005045.1	Cytochrome P450	PHI:438	Reduced virulence
EVM0000369.1	Cysteine-rich secretory protein family	PHI:184	Reduced virulence
EVM0005160.1	Candida agglutinin-like	PHI:527	Reduced virulence
EVM0002221.1	Serine carboxypeptidase	PHI:901	Unaffected pathogenicity
EVM0005124.1	Secretory lipase	PHI:2928	Unaffected pathogenicity
EVM0000326.1	Lipase (class 3)	PHI:2925	Unaffected pathogenicity
EVM0000263.1	DnaJ domain	PHI:1414	Unaffected pathogenicity
EVM0003862.1	DnaJ domain	PHI:1414	Unaffected pathogenicity
EVM0000119.1	GH47	PHI:2510	Unaffected pathogenicity
EVM0004965.1	Hyphally regulated cell wall protein N-terminal	PHI:2599	Unaffected pathogenicity
EVM0001885.1	Hyphally regulated cell wall protein N-terminal	PHI:2599	Effector (plant avirulence determinant)
EVM0002018.1	Hyphally regulated cell wall protein N-terminal	PHI:2599	Effector (plant avirulence determinant)
EVM0003346.1	Hyphally regulated cell wall protein N-terminal	PHI:2599	Effector (plant avirulence determinant)
EVM0003396.1	Hyphally regulated cell wall protein N-terminal	PHI:2599	Effector (plant avirulence determinant)
EVM0003541.1	Hyphally regulated cell wall protein N-terminal	PHI:2599	Effector (plant avirulence determinant)
EVM0003928.1	Hyphally regulated cell wall protein N-terminal	PHI:2599	Effector (plant avirulence determinant)
EVM0003205.1	3-Beta hydroxysteroid dehydrogenase/isomerase family	PHI:325	Effector (plant avirulence determinant)
EVM0003884.1	ERG2 and Sigma1 receptor like protein	PHI:832	chemistry target
EVM0003644.1	Copper/zinc superoxide dismutase (SODC)	PHI:383	Loss of pathogenicity
EVM0000853.1	Receptor L domain	PHI:333	Loss of pathogenicity
EVM0003931.1	Aminotransferase class-III	PHI:3126	Loss of pathogenicity
EVM0004604.1	GH31	PHI:1071	Loss of pathogenicity
EVM0003093.1	–	PHI:2611	Loss of pathogenicity

### Comparative Analysis of Carbohydrate-Active Enzymes

**FIGURE 3:**
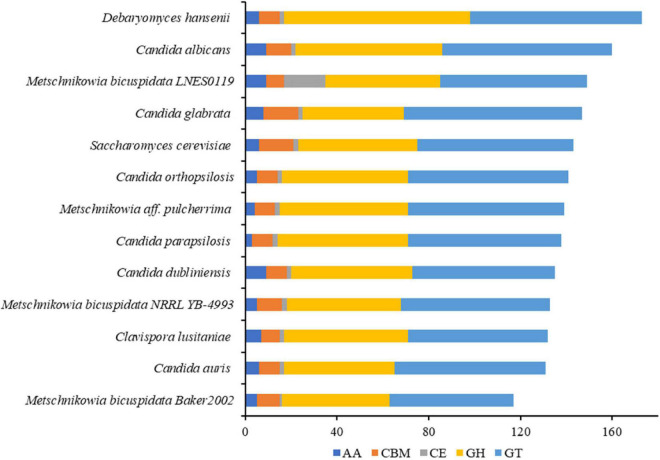
Number of CAZymes genes in *M. bicuspidata* LNES0119 and the other 12 yeasts. GH: glycoside hydrolase, GT, glycosyltransferase; CE, carbohydrate esterase; AA, auxiliary activity; CBM, carbohydrate-binding module.

The numbers of CAZymes in human/animal pathogenic and non-pathogenic yeasts varied between 117 and 173, all of which were without polysaccharide lyases (PLs). The lowest number was in *M. bicuspidata* Baker 2002 and highest in non-pathogen *Debaryomyces hansenii* (Figure 3). The number of CAZymes in *M. bicuspidata* LNES0119 was lower than that in the human pathogen *C. albicans* and non-pathogen *Debaryomyces hansenii* (Figure 3). CBM18, CBM21, CBM43, and CBM48 were present in each fungus, but CBM48 was absent in *M. bicuspidata* LNES0119 (Supplementary Table 6). In addition, *M. bicuspidata* LNES0119 had 18 CE, which was the highest among these yeast genomes. CBM20, CBM23, CE1 (8), CE8, CE10 (6), CE12, CE14, AA3, and AA7 are unique to the *M. bicuspidata* LNES0119 genome (Supplementary Table 6).

### Comparative Genomics of *Metschnikowia bicuspidata* LNES0119 With Other Strains

**FIGURE 4:**
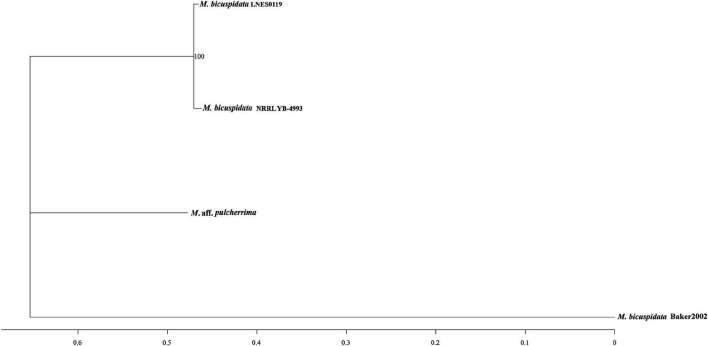
Interspecific phylogenic tree of three *M. bicuspidata* strains and *M.* aff. *pulcherrima*.

**FIGURE 5:**
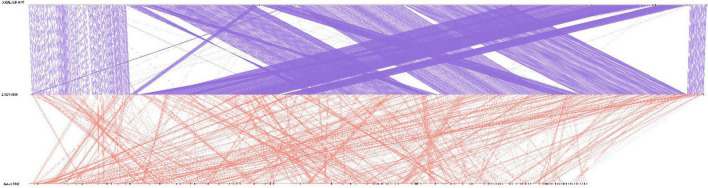
Parallel collinearity comparison between three *M. bicuspidata* strains LNES0119, NRRL YB-4993, and Baker 2002.

As shown in Table 1, the genome size was similar between LNES0119 (16.13 Mb) and NRRL YB-4993 (16.06 Mb); meanwhile, the phylogenetic tree revealed that these two *M. bicuspidata* strains evolved closely, as determined according to their single copy homologous genes (Figure 4). In addition, there were 10,522 (92.52%) collinear genes between *M. bicuspidata* LNES0119 and NRRL YB-4993 strains, whereas there were 6,809 (65.83%) collinear genes between *M. bicuspidata* LNES0119 and Baker 2002 strains (Figures 4, 5). We found that the *M. bicuspidata* LNES0119 and NRRL YB-4993 strains were largely linearly syntenic, but there was a total of six evident inversions between the two assemblies (Figure 5).

Comparative analyses of gene families were conducted among four *Metschnikowia* strains, including three *M. bicuspidata* strains and one non-pathogen, *M.* aff. *Pulcherrima* APC 1.2. We identified 5,411 putative gene families, of which 2,948 homologous families were shared among four *Metschnikowia* species, including 2,785 single-copy genes. There were 82 common family genes present in three pathogenic strains of *M. bicuspidata* that were absent in the non-pathogenic species *M.* aff. *pulcherrima* APC 1.2 (Table 4 and Supplementary Table 7). A total of 331 unique genes were found in *M. bicuspidata* LNES0119, 30 of which were putatively related to pathogenicity, and they were primarily cell wall genes, including eight Hyr/Iff-like genes (Pfam annotation to hyphally regulated N-terminal) as well as one agglutinin-like protein 5 and secreted aspartyl protease (Table 4 and Supplementary Table 8). In addition, only 12 unique gene families were observed in *M. bicuspidata* LNES0119, of which two gene families (GF3117 and GF3118) represented by six genes belonged to the Pfam domain (PF11765.3, hyphally regulated cell wall protein N-terminal) and were putatively associated with pathogenicity (Table 4 and Supplementary Table 9).

**TABLE 4:** Statistics of the gene families of four *Metschnikowia* strains.

Type	Total gene families	Clustered gene families	Shared genes	Unique genes	Unique gene families
*M. bicuspidata* LNES0119	5,088	5,278	5,236	331	12
*M. bicuspidata* Baker 2002	4,942	4,212	4,155	730	24
*M. bicuspidata* NRRL YB-4993	4,101	4,391	4,275	1,810	36
*M.* aff*_pulcherrima* APC 1.2	4,031	5,541	5,199	819	84

## Discussion

The genome of *M. bicuspidata* LNES0119 strain was successively sequenced using Illumina and Oxford Nanopore platforms in combination, and the assembly size of the genome was similar to that of *M. bicuspidata* NRRL YB-4993. The six contigs and 99.3% complete BUSCO estimation demonstrated that the genome was a high-integrity assembly. When compared with that of the three *M. bicuspidata* strains, the homology of *M. bicuspidata* LNES0119 and NRRL YB-4993 strains was found to be higher than that of the Baker 2002 strain in a phylogenetic tree analysis, and this result is consistent with the results of the synteny analysis. Ahrendt et al. (2018) considered that the *Daphnia* parasite *M. bicuspidata* Baker 2002 was unculturable and not conspecific to the brine shrimp parasite *M. bicuspidata* NRRL YB-4993. The two strains of *M. bicuspidata* LNES0119 and NRRL YB-4993 had good coverage with each other, but there were six large segment inversions, which revealed that the two genomes experienced a particular genome structure variation during the process of evolution, which might have led to changes in coding genes and even changes in functional proteins.

CAZymes are responsible for the breakdown, biosynthesis, or modification of glycoconjugates, oligo-, and polysaccharides, and are known to play important roles in host-pathogen interactions (Zhao et al., 2013). In our study, 64 CAZymes of *M. bicuspidata* LNES0119 annotated to PHI are involved in carbohydrate metabolic processes of protein O-linked glycosylation, the glucan catabolic process, and protein mannosylation. Previous studies have demonstrated that these proteins play vital roles in cell wall assembly and construction of human pathogenic yeasts against environmental stress conditions, act as virulence factors in pathogenicity, or cause a cell-mediated host immune response (Chaffin et al., 1998; Stubbs et al., 1999; Chaffin, 2008; Mora-Montes et al., 2009; Hall and Gow, 2013). This suggests that these CAZymes of *M. bicuspidata* LNES0119 might be involved in the composition and maintenance of the cell wall during infection. In addition, comparative analysis of the CAZymes genes of 13 species of human/animal and non-pathogenic yeast showed that they all lacked PLs and the number of CAZy genes varied from 117 to 173, which is far less than that found in the genomes of ascomycete fungi (Gazis et al., 2016; Peng et al., 2017). This distinction in the composition and size of CAZymes was closely related to host specificity and the lifestyle adaptations of the fungi. For example, the number of CAZymes in 97 species of saprophytic, pathogenic, and endophytic fungi varied from 215 to 1,938 (Wan, 2019). Compared to the 94 saprophytic, facultative parasitic, hemi-biotrophic, biotrophic, and symbiotic fungi, there were 12 saprophytic or facultative parasitic species such as yeasts and fungi in the genus *Trichophyton* that lacked PLs (Zhao et al., 2013). By comparing the genomes of pathogenic animal and plant fungi, it was found that specialization to different hosts drives a distinct CAZyme family repertoire (Gaulin et al., 2018). In our study, the lowest number of CAZymes shown in *M. bicuspidata* Baker 2002 might be closely related to its non-free-living lifestyle. It was found that *M. bicuspidata* Baker 2002 was unculturable *in vitro* because it lacked 15 CAZymes that were broadly linked to aspects of urea, sulfate, and thiamine metabolism when compared with that of the NRRL YB-4993 strain (Ahrendt et al., 2018). These results suggest that *M. bicuspidata* Baker 2002 might have a reduced number of CAZymes as a strategy to adapt to the host environment. In a similar way, the reduced number of 30 CAZymes observed in the endophytic fungus *Xylona heveae* may be an adaptation to enable intercellular growth in its rubber tree host (Gazis et al., 2016). However, the CE1 and CE10 gene families were unique in *M. bicuspidata* LNES0119 and the extended role of this gene family is unknown. The enrichment of CE1 and CE10 homologs observed in hemibiotrophic and necrotrophic Oomycetes suggests that they are used either in a species-specific manner during infection or only for pathogens with certain similar characteristics (Gaulin et al., 2018). Taken together, these results might provide insights into the close relationship of CAZymes with the lifestyle and pathogenicity of pathogenic yeasts, which highlights the need for further research.

Fungal lifestyle closely relies on proteins that are secreted extracellularly for growth within their hosts, and these secreted proteins play an important role in mediating interactions with hosts (McCotter et al., 2016). In the *M. bicuspidata* LNES0119 strain, 41 secretory proteins were found to be related to pathogenicity. Of these, cell wall proteins were the most abundant (Table 3). It is well known that the cell wall of pathogenic fungi maintains the integrity of the cell and interacts with the environment; it also plays a major role in the interaction with the host cells by adhering to their surfaces, invading tissues, and protecting the pathogen from host defense mechanisms. Interestingly, we found that cell wall proteins of hyphally regulated N-terminal domains, eukaryotic aspartyl protease domains, and the *Candida* agglutinin-like domain were unique to *M. bicuspidata* LNES0119 (Supplementary Table 8); however, these cell wall proteins were also found to be highly enriched in the strong human pathogens *C. albicans*, *C. tropicalis*, and *C. parapsilosis* (Butler et al., 2009). In these *Candida* spp., these proteins have been confirmed to be closely associated with pathogenicity and virulence by primarily participating in adhesion to host surfaces and invasion of host cells (Bailey et al., 1996; De Bernardis et al., 2001; Naglik et al., 2003; Butler et al., 2009; de Groot et al., 2013). Our results imply that the roles of unique cell wall proteins in *M. bicuspidata* LNES0119 are similar to those of human pathogens during infection. Furthermore, eight secreted proteins in *M. bicuspidata* LNES0119 were shown to be hydrolytic proteins, including lipases, proteases, and phospholipases (Table 3). Previous studies have demonstrated that fungal hydrolytic enzymes (lipases, proteases, and phospholipases) as virulence factors increase the pathogenicity to insects and humans (Hruskova-Heidingsfeldova, 2008; Gaillardin, 2010; Bentubo and Gompertz, 2014; Mondal et al., 2016; Petrisor and Stoian, 2017). For human *Candida* species, these hydrolytic enzymes can contribute to their invasion of the host tissue through digestion or destruction of the cell membrane, help the pathogen avoid the host defense immune system, and allow the microorganism to utilize host cell macromolecules as a source of nutrients (Schaller et al., 2005; Hruskova-Heidingsfeldova, 2008). For entomopathogenic fungi, hydrolytic enzymes are primarily involved in the initial stages of the adhesion and penetration of insect cuticles (Petrisor and Stoian, 2017). Thus, it was reasonable to infer that these hydrolytic proteins of *M. bicuspidata* LNES0119 are involved in adaptation and pathogenicity to their host. Finally, other secreted proteins, such as HSP70, multicopper oxidase, and thioredoxin might have taken part in the process of thermal adaptation, nutrient acquisition and stress tolerance, which have also been detected as virulence factors involved in the pathogenicity of fungi (Zhu and Williamson, 2004; Puri and Edgerton, 2013; Chakraborty et al., 2020; Wang et al., 2020). Further research should focus on these proteins because they might play a vital role in the regulation of crab–pathogen interactions.

## Conclusion

In the present study, high-quality assembly and complete genome analysis were performed in *M. bicuspidata* LNES0119. Genomic and comparative analyses revealed that the genome of *M. bicuspidata* LNES0119 possesses a variety of putative pathogenic genes, which are primarily involved in cell wall assembly and construction and might play a vital role in adapting to the host environment or acting as virulence factors in pathogenicity or causing a cell-mediated host immune response. Therefore, these candidate factors provide a novel resource for further study of the pathogenic mechanisms in *M. bicuspidata*–associated diseases as well as for the identification of potential targets for further research and therapeutic intervention.

## Data Availability Statement

The datasets presented in this study can be found in online repositories. The names of the repository/repositories and accession number(s) can be found below: https://www.ncbi.nlm.nih.gov/, PRJNA803590.

## Ethics Statement

The animal study was reviewed and approved by the Animal Experiments Ethics Committee of Shenyang Agricultural University.

## Author Contributions

HJ and QC conceived and designed the project. JB, HJ, and YX prepared the strain samples and conducted the bioinformatics analysis. HJ, JB, XL, and QC wrote the manuscript. All authors contributed to the article and approved the submitted version.

## Conflict of Interest

The authors declare that the research was conducted in the absence of any commercial or financial relationships that could be construed as a potential conflict of interest.

## Publisher’s Note

All claims expressed in this article are solely those of the authors and do not necessarily represent those of their affiliated organizations, or those of the publisher, the editors and the reviewers. Any product that may be evaluated in this article, or claim that may be made by its manufacturer, is not guaranteed or endorsed by the publisher.

## Footnotes

1
http://transdecoder.github.io
