# Supplementary material for: Comparative Genomic Analyses Provide Insight Into the Pathogenicity of Metschnikowia bicuspidata LNES0119
Source: Front Microbiol. 2022 Jun 13;13:939141. doi: 10.3389/fmicb.2022.939141 (PMC9234493; doi:10.3389/fmicb.2022.939141)
Supplement: Supplementary file 1 [file Data_Sheet_1.docx]

***Supplementary Figures***


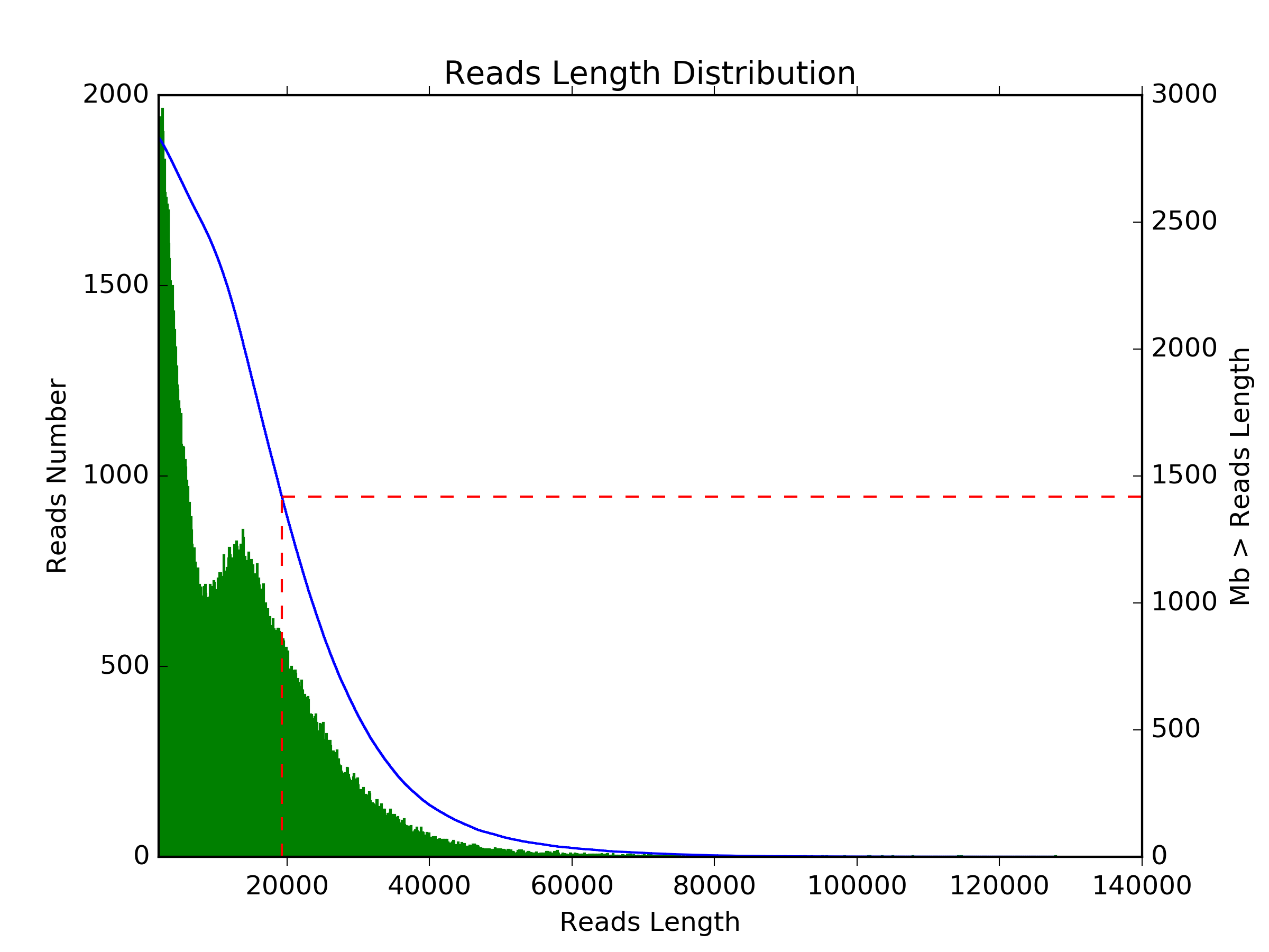


**Supplementary Figure 1.** Length distribution of long and high-quality reads. A total of 2,930,618,879 bp raw data and 2,836,556,465 bp clean reads with estimated genome coverage of 175.28 X were obtained. The x-axis represents read length. The y-axis on the left represents the numbers of reads with specific length, shown by the green bar chart. The y-axis on the right represents the total numbers of bases (Mb) contained in reads larger than specific length, shown by the blue curve. The red dotted line represents the N50 length of reads.


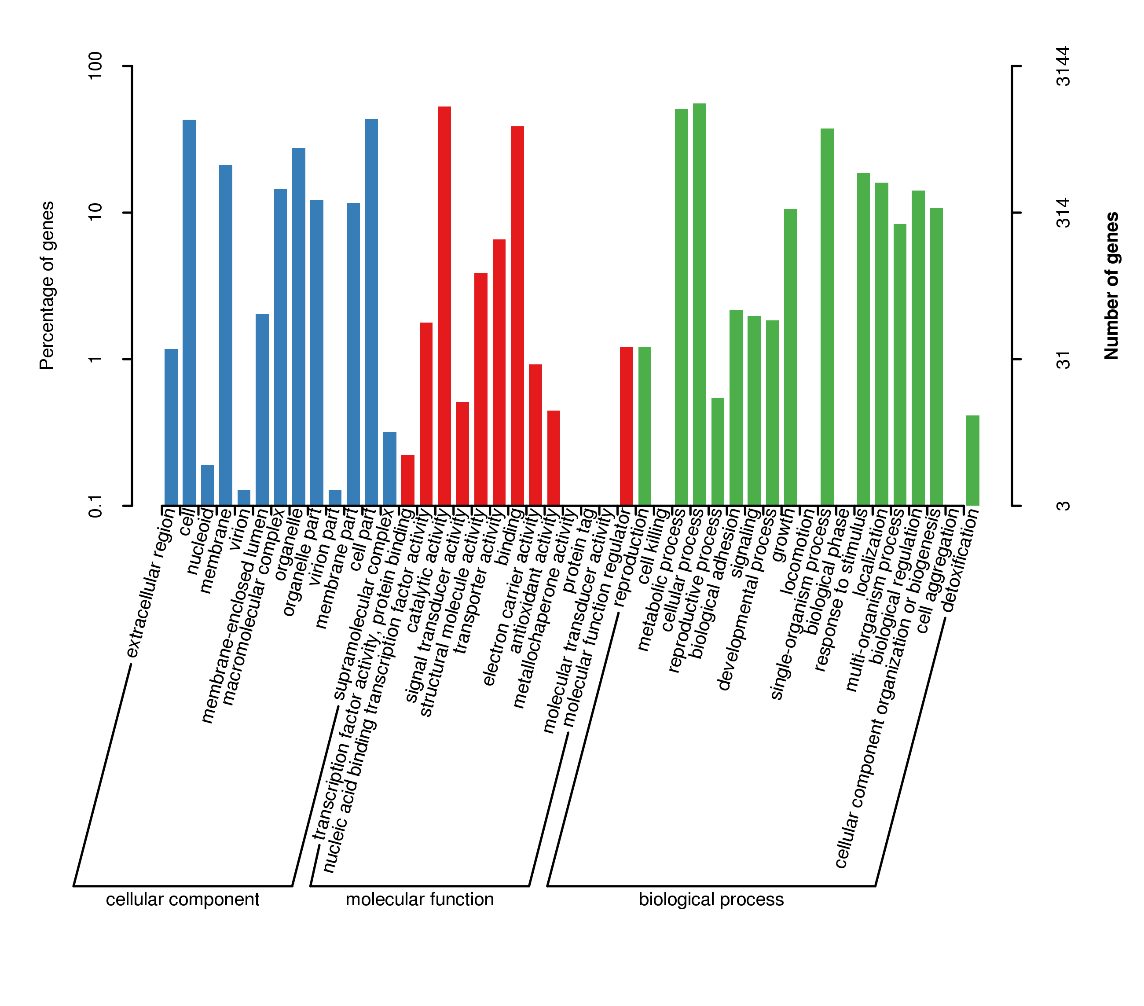
 **Supplementary Figure 2.** Gene Ontology (GO) functional annotation of the *Metschnikowia bicuspidata* LNES0119. All the genes are classified into three categories: biological processes (BP), cellular components (CC), or molecular functions (MF). The X-axis represents various gene functions and the Y-axis corresponds to the percentage of genes and gene number.


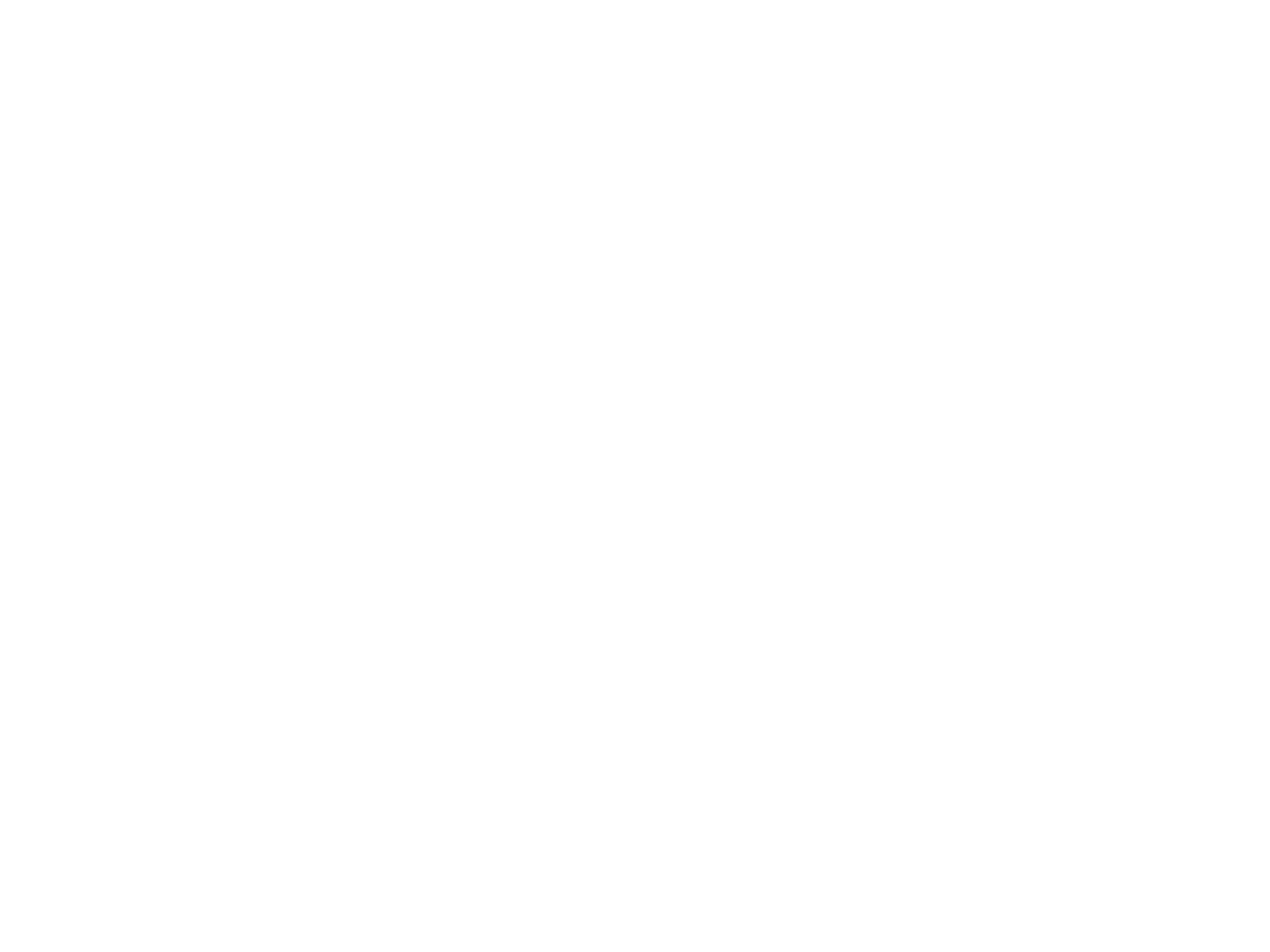


**Supplementary Figure 3.** The Kyoto Encyclopedia of Genes and Genomes (KEGG) function annotation of the *M. bicuspidata* LNES0119 genome.
